# A Dotted *Triangle* or Dots of *Three*: The Role of Representational Content on Working Memory Capacity in Early Childhood

**DOI:** 10.1111/desc.70066

**Published:** 2025-08-18

**Authors:** Tongyan Ren, Xuechen Ding, Chen Cheng

**Affiliations:** ^1^ School of Psychology Shanghai Normal University Shanghai China; ^2^ Shanghai Experimental School Shanghai China; ^3^ Lab for Educational Big Data and Policymaking Ministry of Education, P. R. China Shanghai China; ^4^ The Research Base of Online Education for Shanghai Middle and Primary Schools Shanghai China; ^5^ Division of Social Science The Hong Kong University of Science and Technology Hong Kong SAR China

**Keywords:** content representation, memory load, preschoolers, working memory storage

## Abstract

Working memory (WM) is a critical cognitive system that supports processing a variety of information. Remembering different types of objects may impose different levels of cognitive demands on WM performance. In the present study, we examined 205 children's WM in representing different types of content and its developmental trajectories in early childhood. Experiment 1 examined 5‐year‐olds’ WM performance when remembering different content (animals and dots). To control perceptual differences, Experiment 2 compared the same‐age children's WM performance when the stimuli (e.g., three dots) needed to be encoded from different representational domains (perceptual domain: visuospatial representation; conceptual domain: numerical representation). In Experiment 3, we further investigated the early developmental trends of representing different types of information in WM between the ages of 3 and 5. Results showed that children's WM performance varied over different types of stimuli. When presented with the same stimuli, encoding different aspects of the content (conceptual vs. perceptual) may impose different levels of cognitive demands, and the performance of which was dependent on the WM loads. Together these findings informed our understanding of the role of representational content in children's WM development and provided empirical implications for considering the testing stimuli when designing WM measurements for young children.

**Summary:**

- Children's working memory (WM) performance varies depending on the type of representational content (conceptual vs. perceptual).
- Encoding the same stimuli from different representational domains imposes varying memory loads in preschool‐aged children.
- Developmental trends in WM for different types of content emerge between ages three and five.
- Findings provide insights for designing assessments tailored to young children's developmental and representational capacities.

## 1 Introduction

“*There are a thousand Hamlets in a thousand people's eyes*”

—‘Hamlet’ by Shakespeare

As the saying goes, each person may interpret *Hamlet* from a unique perspective based on their own ideas, experiences, and thoughts. Similarly, when we perceive the same object, not everyone constructs the same mental representation. This variability may help explain the individual differences observed between laypeople and those with professional expertise. For instance, chess players can quickly and accurately recall chessboard configurations within seconds (Chase and Simon 1973), whereas untrained individuals perceive the same configurations as a confusing puzzle. While such expertise takes years to develop, even in early childhood, young children may encode different aspects of an object into working memory (WM), leading to varied memory outcomes.

To remember information in support of ongoing tasks, WM is the cognitive system that temporarily holds and manipulates active mental representations, making them accessible for processing during ongoing tasks (Baddeley and Hitch 1974; Logie and Cowan 2015). It is crucial for cognitive development in children (Bayliss et al. 2003; Holmes et al. 2010), as it supports a wide range of cognitive functions that are essential for academic and social success (Bull et al. 2008; Cowan 2014, 2016; Geary 2011; Gray et al. 2019, 2017; Jarrold and Towse 2006; Menon 2016).

The capacity to remember objects in WM develops rapidly in early childhood in multiple dimensions (e.g., Cowan et al. 2006, Cowan et al. 2010; Káldy and Blaser 2013; Káldy and Leslie 2003, 2005; Ross‐Sheehy et al. 2003; Gathercole et al. 2004; Isbell et al. 2015; Riggs et al. 2006, 2011). From 7 months, the number of items that infants can encode into their WM increases from one to 3–4 items by the end of the first year, depending on the tasks (e.g., Feigenson and Carey 2005; Káldy and Leslie 2005; Ross‐Sheehy et al. 2003). This rapid improvement is also accompanied by infants’ growing representational precision in remembering objects with multiple features (Simmering et al. 2015). In infancy, visual WM tasks often focus on single perceptual features, such as color or size, to help individuals distinguish between objects (Diamond et al. 1997). For example, studies focusing on young infants usually used circles and triangles as experimental stimuli in WM tasks (e.g., Káldy and Leslie 2003, 2005; Kibbe and Leslie 2011). Later, with the increase in WM capacity and the maturation of attentional control, children can process more complex stimuli by integrating multiple features (Alvarez and Cavanagh 2004; Cheng et al. 2020; Luck and Vogel 1997; Xie et al. 2025) and from multiple modalities simultaneously (Cowan and Guitard 2024). With age, children also shift from focusing on a single dimension to incorporating a broader range of features, moving from perceiving objects based on appearance to understanding their function and social significance (Carey 2009; Baillargeon et al. 2012).

The extent to which specific features are processed in WM can be influenced by the types of information being remembered, such as perceptual information (e.g., visual and spatial features) or conceptual information (e.g., categories) (Brady et al. 2016). Perceptual features refer to the physical attributes of objects, such as color, size, and shape, whereas conceptual features involve the meaning, function, or category of an object (Cowan 2014; Simmering et al. 2015). These two types of features may be processed and stored differently within the WM system, leading to varying WM performance during early childhood, a period when the conceptualization of objects and the world is still developing.

Perceptual features are typically remembered more quickly, as they are relying on direct sensory input and are processed primarily in the occipital cortex (Brady et al. 2013; Ishai et al. 2000). In contrast, conceptual features are more complex and require higher levels of cognitive processing, such as abstract thinking and categorization, which are not yet fully developed in early childhood (Baillargeon et al. 2012). Research by Oberauer and Eichenberger (2013) demonstrated that color is the most easily remembered feature, followed by shape, size, and orientation. Similar patterns have been observed in children, particularly between ages 3 and 7 (Simmering et al. 2015).

With the experience and knowledge accumulated from daily interaction, children begin to understand the categories of objects and the relationships between different objects, which allows them to integrate not only perceptual features but also conceptual information into their WM representations (Carey 2009; Cowan 2014; Feigenson and Halberda 2008; Kibbe and Leslie 2019; Zosh and Feigenson 2012). Conceptually rich stimuli help children organize stimuli into different groups with hierarchies. For instance, when presented with stimuli that naturally fall into conceptual categories (e.g., a red round circle with texture may be categorized as “balls,” and a brown fluffy body with four extensions may be categorized as “dogs”), infants can use this conceptual information to organize and classify the information to be remembered, thereby saving up WM resources (Feigenson and Halberda 2008).

Stimuli like familiar or meaningful objects can activate long‐term memory, which can enhance the recall of features of objects in WM (e.g., Chung et al. 2023) and therefore expand the WM storage capacity (e.g., Brady et al. 2016). For example, certain combinations of letters, like NBA, may be easier to remember due to the conceptual information embedded within these letters, invoking a meaningful context or pattern that aids in their information processing in WM. As children grow, their ability to remember more objects and features improves (Forsberg et al. 2023; Clark et al. 2018). This enhanced memory performance is often attributed to the representation of conceptual rather than perceptual content, with categorical identity being more easily remembered than specific, detailed features (Kibbe and Leslie 2019).

While growing research suggests that different types of stimuli may affect WM performance. No studies have systematically compared children's WM performance when remembering different types of stimuli across early development. One challenge is that objects with conceptual complexity (e.g., faces, scenes) are often confounded with perceptual complexity, making it hard to identify the developmental sources that are related to the variations in performance. Therefore, it is crucial to disentangle perceptual complexity from conceptual complexity to accurately examine the representational precision of WM in processing different types of information.

In young children's learning environment, sets of items, such as discrete objects, are commonly used in the non‐symbolic format of early numerical learning (e.g., Chen et al. 2023; Cheng and Kibbe 2025; Kibbe and Feigenson 2017). However, no studies to date have directly examined what kind of representations children hold for sets of dots in WM. For instance, whether their representation of sets is equivalent to their representations of individual objects? Since sets of dots can be remembered as numerical entities and/or as pure visual configurations with specific spatial properties, examining how children remember sets of dots using conceptual and/or perceptual information in WM could provide valuable insights into the extent to which different representational domains influence children's memory performance. In the current study, we used sets of dots as one kind of experimental stimuli to examine this question.

## 2 Current Study

The present study aims to examine children's representational precision in remembering different types of information in WM. First, in order to examine whether memory content affects children's WM performance, in Experiment 1, we asked 5‐year‐old children to remember two types of stimuli in WM: animal images, which contain complex perceptual features and rich conceptual information (e.g., “cat”), and images of sets of dots, which contain simple perceptual features and rich conceptual information (e.g., “one”). In Experiment 2, to minimize the effects caused by different levels of perceptual complexity, we controlled the perceptual complexity and compared children's WM performance when remembering different types of information extracted from the stimuli: perceptual information (e.g., “dots that aligned to be a triangle”), which taps the visuo‐spatial representation, or conceptual information (“three dots”), which taps the numerical representation. We hypothesized that in both experiments, children's WM performance will differ depending on the types of stimuli and representational content. Specifically, children's WM performance in remembering conceptual‐rich items will be better than in remembering items that contain poor conceptual information.

To examine the early developmental trends of children's WM ability to remember different types of representational content across perceptual and conceptual dimensions in early childhood, in Experiment 3, we tested younger children's WM performance using the four WM tasks previously tested in Experiments 1 and 2. We extended the age range to include children as young as 3 years old, aiming to identify emerging differences in how conceptual and perceptual information is represented in WM during early childhood.

## 3 Experiment 1

### 3.1 Method

#### 3.1.1 Participants

Forty 5‐year‐old Chinese preschoolers participated in this study (*M*_age_: 68 months, range: 62–71 months, 20 girls). All children were identified as Han ethnicity; 77.5% of the parents held a bachelor's or higher degree. The sample size is large enough to detect a small to medium effect size (*f* = 0.20) in a repeated measure ANOVA with two factors (Set Size: 2, 3, and 4; memory content: animal vs. dots), requiring a sample size of *n* = 28 to achieve 80% power. The data collection was conducted in a kindergarten in Shanghai, China. The study protocol was approved by the ethical committee of the Shanghai Normal University.

#### 3.1.2 Stimuli and Apparatus

**FIGURE 1:**
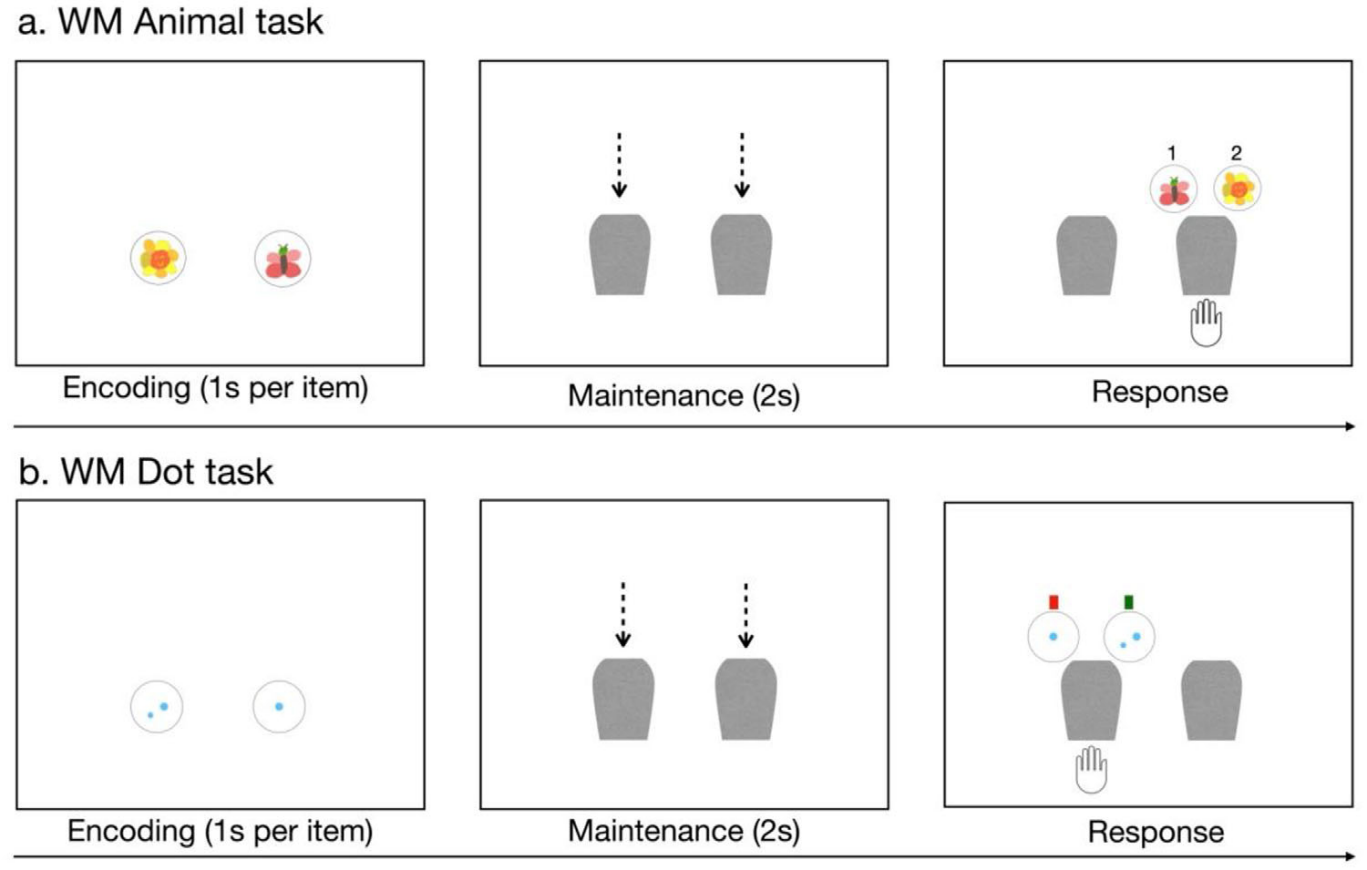
Sample test trials of Experiment 1. The top panel shows a test trial in WM Animal task, and the bottom panel shows a test trial in WM Dot task.

Testing materials were presented using a laptop with a Windows system and on a 13.4‐inch screen. All stimuli were displayed using the Windows Media Player software. Stimuli displayed on the virtual cards were animal images that were adopted from the World of Eric Carle Mini Memory Match Game (Mudpuppy Toys) (see Figure 1a) (Animal WM task) and 1–4 dots (Dot WM task) (experimental material has been uploaded on OSF: https://osf.io/m4fbq).

#### 3.1.3 Design

The experiment adopted a within‐subject design. All participants completed two WM tasks measuring children's WM capacity with different representational content. In the WM Animal task, colorful animal images were displayed on the face of the virtual cards. In the WM Dot task, sets of dots were used. The number of dots appearing on each of the virtual cards was based on previous work showing that preschool children can subitize three to four items within a small set of objects (e.g., Chi and Klahr 1975; Gelman and Tucker 1975; Starkey and Cooper 1995). Therefore, in our task, we included images consisting of one, two, three, or four dots on each virtual card. Both WM tasks followed the same procedures as in previous studies (Cheng and Kibbe 2022, 2024). The order of whether children completed the WM Animal task or the WM Dot task first was counterbalanced across participants.

#### 3.1.4 Procedure

Children were invited to participate in the study individually with a female experimenter in a corner of a quiet classroom in the kindergarten. After a brief introduction, children were asked to play “hide‐and‐seek” games with the experimenter.

##### 3.1.4.1 WM Animal Task

The WM Animal task followed the same procedure as the WM storage task used in the study by Cheng and Kibbe (2022). The use of the same stimuli served the purpose of replicating previous findings in American children. A schematic of a trial is shown in Figure 1a. During the practice trial, children were presented with two cards for 2 s, each displaying a different animal image. The cards were then occluded by flying blocks for 1 s. After an additional 1 s, a virtual hand appeared below one of the cards, with two options matching the previously displayed images shown above the probed location, each labeled with a number (1 or 2). The experimenter instructed children to identify the animal hidden behind the probed location by either stating the corresponding number above the animal option or pointing to the animal option directly. After children responded, the experimenter provided children with feedback on the correctness of their answer. If the response was incorrect, children were given a second chance to complete the trial.

During the test session, children completed test trials following the same procedures as in the practice trials, but without feedback. The session began with two cards displaying different animals, and the number of cards gradually increased to three, four, five, and six cards. The encoding time varied depending on the number of cards to be encoded, with 1 s allocated per card. Thus, when two cards were displayed on the screen, the encoding time was 2 s, and when three cards were displayed, the encoding time was 3 s. The maintenance time was kept consistent with the practice trial, during which flying cards moved downward to occlude the cards for 1 s. After an additional 1 s, a virtual hand was revealed. There was no time limit during the response period, and the trial ended once the child provided a response. Each set size included four trials, resulting in a total of 20 test trials. The task lasted about 12–15 min.

##### 3.1.4.2 WM Dot Storage Task

The procedure and presentation timing were identical to the WM Animal task, except that the animal images were replaced with dot patterns. Children first completed two practice trials. In the practice session, children were shown two cards, each displaying a different number of dots. After a brief display, occluders descended to cover the cards. A virtual hand then probed one of the cards, and the two previously displayed cards reappeared above the probed card. Two color bars—red and green—appeared above the two option cards. Children were encouraged to find out the image of the probed card by choosing between the two options. They were asked to either state the corresponding color bar (red or green) next to the correct card or point to it directly (see Figure 1b). Feedback was provided by the experimenter, following the same procedure as in the WM Animal task.

The test trial proceeded similarly to the practice sessions. However, to ensure the task remained within children's subitizing range—the ability to quickly and accurately identify small quantities—set sizes were limited to a maximum of four (e.g., Chi and Klahr 1975; Gelman and Tucker 1975; Starkey and Cooper 1995). The number of dots on the cards ranged from one to four. Children completed four test trials for each set size, resulting in a total of 12 test trials. The task took approximately 7–10 min to complete.

The WM Animal task and the WM Dot task were made the same in the procedures except for the to‐be‐remembered stimuli. There was also a small difference during the response period. If children chose to respond verbally, they needed to say the corresponding number (1 or 2) next to the card option in the WM Animal task and the corresponding color (red or green) next to the card option in the WM Dot task (see Figure 1). Since children at this age can recognize the number “1” or “2” and the color “red” or “green” with no problems (we have also ensured that they understood the instructions during practice trials), selecting and saying the number or the color should not pose additional cognitive differences to children during responses.

##### 3.1.4.3 Coding

Children's responses for each trial were coded as 1 (correct) or 0 (incorrect). Their performance was then averaged over set sizes and tasks for further analyses.

### 3.2 Results

To determine whether children were able to perform different WM tasks, we first conducted one sample *t*‐tests and compared children's averaged proportion correct in each condition for different set sizes to chance level (0.50). We set the alpha value to determine the statistical significance to 0.001 in WM Animal task and 0.0167 in WM Dot task to correct for multiple comparisons within each task. Results showed that children's performance was significantly above chance when remembering up to five items in the WM Animal task and up to four items in the WM Dot task (all *t*(39) > 3.74, *p* < 0.001, Cohen's *d* > 1.19). Children's performance in remembering six items in the WM Animal task was at chance (*t*(39) = 1.35, *p* = 0.092, Cohen's *d* = 0.43) (see Supporting Information: Table S1 for full details of the statistical results).

**FIGURE 2:**
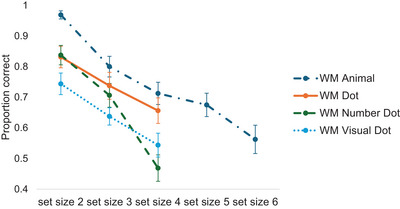
Combined results of Experiments 1 and 2. The *y*‐axis shows the proportion correct of children's performance in each WM task. The *x*‐axis shows set sizes. The dashed dot shows performance in the WM Animal task; the solid line shows performance in the WM Dot task; the dashed line shows performance in the WM Number Dot task; and the dotted line shows performance in the WM Visual Dot task. Error bars indicate standard error.

Next, we examined whether children's memory performance was influenced by memory loads and memory content. We conducted a generalized logistic regression model using the lme4 package in R (Bates et al. 2014) and included task type (WM Animal task and WM Dot task) and set size (Set Size 2, 3, 4) as independent variables and set Participant and Trial number as random intercepts to examine these effects on children's memory performance. The best‐fitting model does not include the interaction. We observed a main effect of task type, *F*(1, 916) = 12.13, *p* < 0.001, *η_p_* ^2^ = 0.01, with children performing better when remembering animal images compared to dotted images. We also observed a main effect of set size, *F*(2, 916) = 26.18, *p* < 0.001, *η_p_* ^2^ = 0.05, with children's memory performance decreasing as the set size increased (see Figure 2).

### 3.3 Discussion

Findings of Experiment 1 revealed that children demonstrated better memory performance when the stimuli were animals with familiar characters, compared to dots with different sizes and spatial orientations. This suggested that the types of content that are held in WM may affect the representational precision of WM. Additionally, we also observed that children's memory performance decreased as the WM loads increased, providing converging evidence to the previous literature (Cheng and Kibbe 2022; Cowan et al. 2010; Ren et al. 2025; Simmering 2012).

What accounts for the discrepancies in WM performance between different types of memory content? One advantage of using animal images in the WM task is the rich conceptual representation embedded in the stimuli. This allows children to automatically integrate conceptual information into the WM processes of these images. For instance, instead of representing the images as “yellow round thing with eyes,” children can easily extract the conceptual meanings and represent items as “cat.” Similarly, the stimuli in the WM Dot task are semantically meaningful, enabling children to extract the numerical meanings and represent sets as “one” or “two.” However, the simplicity of the perceptual features in the dot stimuli may introduce more noise into children's representation. This could make it more challenging for children to distinguish between sets, as their processes might also rely on the low‐level features, which prevented the formation of deeper, higher‐level representations that are more distinct and less susceptible to variability (Brady and Störmer 2022; Starr et al. 2020).

To disentangle the effect of the drastic perceptual differences between the two types of stimuli used in Experiment 1, the next experiment investigated children's representation of similar objects when different types of information (conceptual vs. perceptual) were extracted for the processes in WM. We hypothesized that, with equivalent perceptual complexity, differences in WM performance between the two tasks would result from the type of information—conceptual or perceptual—being represented in WM.

## 4 Experiment 2

### 4.1 Method

#### 4.1.1 Participants

The same children (*n* = 40) from Experiment 1 participated in Experiment 2 during a separate visit, scheduled within 2 weeks of the first session in the same semester. This sample size was sufficient in detecting a small to medium effect size in a repeated measure ANOVA with two factors (Set Size: 2, 3, and 4; memory content: visual and numerical) (*f* = 0.20, power = 80%), requiring a total of 28 participants. All children participated in the second visit.

#### 4.1.2 Apparatus and Stimuli

The same apparatus and stimuli were used in Experiment 1.

#### 4.1.3 Design

**FIGURE 3:**
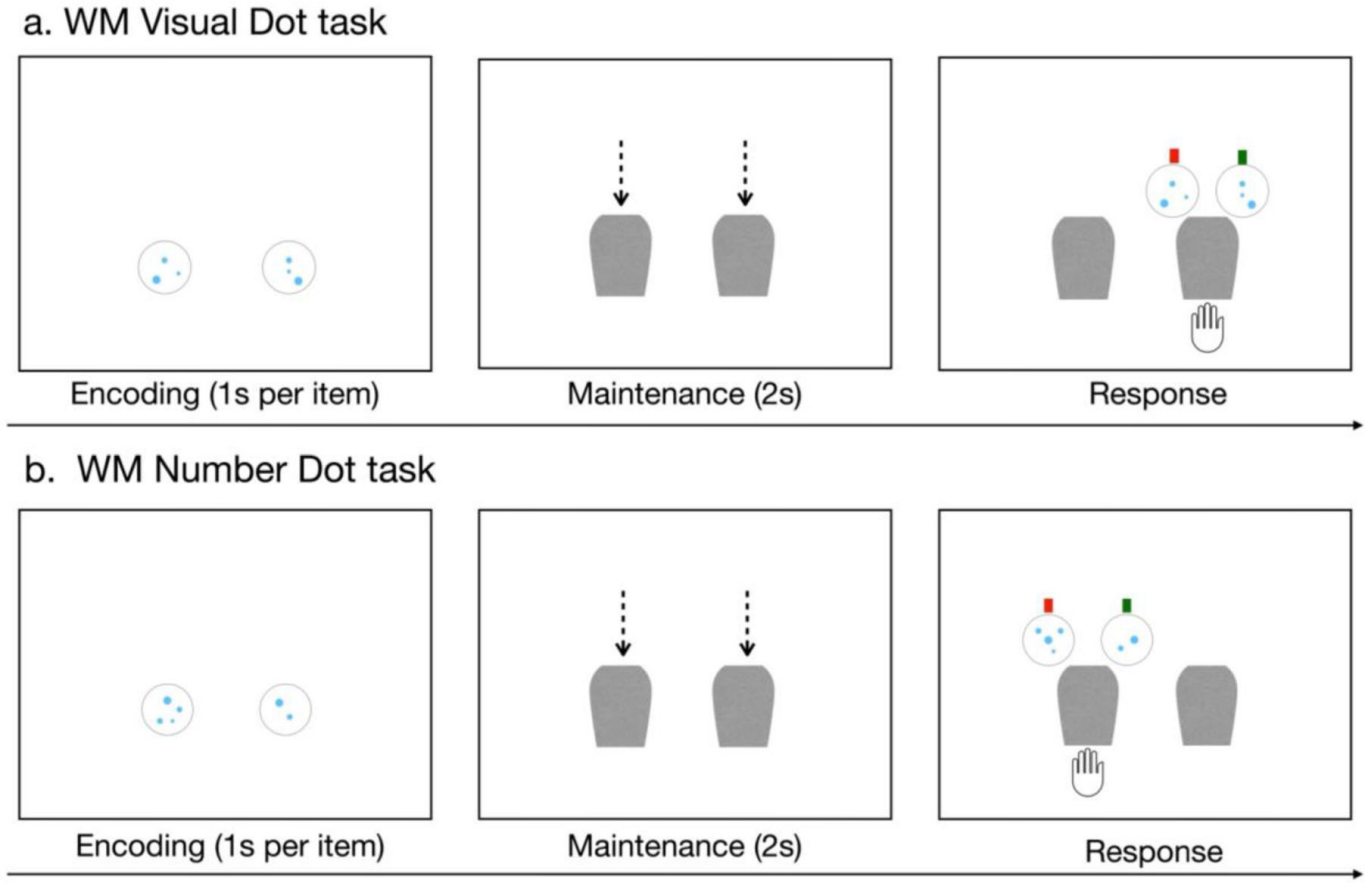
Sample test trials of Experiment 2. In each trial of the WM Visual Dot task, all cards displayed the same number of dots but with varying visuo‐spatial layouts, requiring children to focus on the perceptual aspects of the dot arrangements. In each trial of the WM Number Dot task, the cards displayed different number of dots that needed to be remembered. During the response period, the given options for a randomly probed card shared only the numerical information from the previously displayed cards, while differing in the visuo‐spatial layouts, encouraging children to focus on the conceptual aspects of the dot sets.

Children completed two WM tasks that were designed to measure their WM capacity in remembering images of dots: the WM Visual Dot task and the WM Number Dot task. The WM Visual Dot task was used to examine children's WM capacity in remembering the perceptual information of different sets of dots. In each trial, all cards showed the same number of dots, thus sharing identical numerical information, but varied in sizes and spatial configurations (see Figure 3a). To distinguish between the cards and accurately identify the probed card, children needed to process the perceptual information, including the dots’ configural orientation and spatial layout.

In contrast, the WM Number Dot task was used to examine children's WM capacity in remembering numerical information of different sets of dots. Children were asked to remember a number of cards displaying sets of dots in varying quantities. In contrast to Experiment 1, during the response period, the two optional cards surrounding the probed card that required children to choose from only shared the same numerical information (e.g., “two” and “four”) as the previously displayed cards, but the visuo‐spatial layouts were different (see Figure 3b). To correctly identify the probed card, children needed to process the conceptual information (e.g., sets of “four” dots), rather than relying on the visuo‐spatial features of the dot arrangement.

#### 4.1.4 Procedure

##### 4.1.4.1 WM Visual Dot Task

The procedures and presentation timing of the WM Visual Dot task were the same as in the WM Dot task. In the practice session, children saw two cards with the same number of dots (e.g., two dots) but different spatial orientations. During the response period, children were asked to find out “which card hid here?” at the probed location (see Figure 3a). Children completed two practice trials and 12 test trials. The task took about 12–15 min to complete.

##### 4.1.4.2 WM Number Dot Task

In the practice session, children were presented with two cards on each trial and asked to identify the correct number‐matching card from two alternatives. During the response period, children were asked to find out “which card has the same number of dots” as the probed, occluded card. After completing three practice trials with feedback, children proceeded to the test session with no feedback. In the test session, children completed a total of 12 test trials to assess their ability to remember sets of two, three, or four cards, each displaying a different number of dots (see Figure 3b). The same presentation timing was used in the WM Number Dot task. The order in which children completed the WM Visual Dot task first or the WM Number Dot task first was counterbalanced across participants. The duration of this task was 12–15 min.

### 4.2 Results

We first conducted one‐sample *t*‐tests to determine whether children can perform the two WM tasks. The alpha value to accept statistical significance was set to 0.0167 to correct for three multiple comparisons within each condition. Results showed that children's performance in Set Sizes 2 and 3 was above chance level for both the WM Number Dot task and the WM Visual Dot task (all *t*(39) > 4.87, *p* < 0.001, Cohen's *d* > 1.56). Children's performance in Set Size 4 was at chance in both the WM Number Dot task (*t*(39) = 0.73, *p* = 0.47, Cohen's *d* = 0.23) and the WM Visual Dot task (*t*(39) = 1.35, *p* = 0.19, Cohen's *d* = 0.43) (see Supporting Information: Table S2 for full details of the statistical results).

Next, we examined whether children's performance was affected by memory load and memory content. Generalized logistic regression was conducted by entering task type and set size as predictors and setting Participant and Trial number as random intercepts. The best‐fitting model included an interaction between task type and set size. We observed a main effect of set size (*F*(2, 916) = 32.25, *p* < 0.001, *η_p_* ^2^ = 0.07); children's performance in both tasks decreased as more items needed to be remembered. An interaction effect between task type and set size was observed (*F*(2, 916) = 3.28, *p* = 0.038, *η_p_* ^2^ = 0.0071). An inspection of Figure 2 showed that while children's performance in the WM Number Dot task was better in Set Size 2 trials, the advantages diminished when more items needed to be remembered. This suggested that children's WM capacity in remembering different aspects of information was dependent on the number of items to be remembered; children's WM representation when remembering the conceptual aspect of the information was as error‐prone as remembering the perceptual aspect of the information. No main effect of task type was observed (*F*(1, 916) = 1.01, *p* = 0.32, *η_p_* ^2^ = 0.001).

Finally, combining data from Experiment 1, we examined whether children's performance in different WM tasks employed similar cognitive resources and performed correlation between their averaged memory performance over set sizes (Table 1). We found that children's memory performance in the WM Animal task and the WM Dot task was positively correlated with all the other WM tasks (all *r* > 0.359, *p* < 0.023), revealing a relatively consistent within‐subject stability in performing different WM tasks. Interestingly, children's performance in the WM Number Dot and the WM Visual Dot tasks was not related to each other (*r* = 0.260, *p* = 0.105). All the effects remained the same after controlling for age (see Supporting Information: Table S3 for details).

**TABLE 1:** Correlational results of children's WM storage performance.

	WM Animal task	WM Dot task	WM Number Dot task
WM Animal task	—	—	—
WM Dot task	0.497^**^	—	—
	(0.001)		
WM Number Dot task	0.359^*^	0.563^**^	—
	(0.023)	(< 0.001)	
WM Visual Dot task	0.476^**^	0.380^*^	0.260
	(0.002)	(0.016)	(0.105)

*Note*:

** *p* < 0.01,

* *p* < 0.05.

### 4.3 Discussion

The results of Experiment 2 provide further empirical evidence on how different types of information—conceptual versus perceptual—are processed in children's WM. We compared children's ability to remember sets of dots when representing either the numerical information of dots or the visuo‐spatial configurations of the dots. Distinct differences emerged in WM performance. Children demonstrated better performance on the WM Number Dot task at smaller set sizes, indicating that focusing on the conceptual and numerical aspect of the to‐be‐remembered dot sets is more efficient and enhances WM performance. This aligns with previous findings emphasizing the role of conceptual knowledge in facilitating the organization of information into structured, meaningful categories, enabling better memory performance through chunking strategies (Carey 2009; Miller 1956; Feigenson and Halberda 2008). However, as the set size increased, the advantage of representing the conceptual information diminished. In the WM Visual Dot task, children relied on the visual spatial features to distinguish and identify different cards with dots. This appeared to be more challenging to children, as evidenced by the lower performance compared to that of the WM Number Dot task.

The interaction between set size and task type suggests that children's WM performance is modulated not only by the amount of information they need to store but also by the type of memory content. In trials with smaller set sizes, children performed better on the conceptual task than on the perceptual task. However, this advantage diminished with larger sets, where performance declined to similar levels for both tasks. This indicates that while conceptual information is more easily remembered under lower cognitive loads, with the increase in WM loads, even remembering the conceptual information can become challenging. Overall, the findings imply that representing the conceptual information of the stimuli may be more beneficial in WM, even though this advantage is constrained by the memory loads.

The stimuli used in the WM Number Dot task and the WM Visual Dot were intentionally designed to facilitate children to attend to either the conceptual (numerical) aspect of the sets or the perceptual (visual spatial) aspect of the sets. Remembering the other kind of information (e.g., remembering the conceptual features of sets in the WM Visual Dot task) would not help children to perform better in the task. For example, in the WM Visual Dot task, it is possible that children may still represent the conceptual dimension—numerical information—of the dot sets. However, since in each trial, all the to‐be‐remembered sets of dots share the same numerical information (e.g., all have three dots; see Figure 3), the conceptual information cannot be used as an effective memory cue. Similarly, in the WM Number Dot task, it is possible for children to remember the visual spatial properties of the dots; however, since the card options provided in the response period did not match the exact visual spatial properties of the dots during the encoding period, remembering the perceptual information would not bring additional benefits in correctly choosing the target. The correlational analyses also provided further evidence supporting this. While performance in the WM Number Dot and Visual Dot tasks was separately correlated with both the WM Animal task and the WM Dot task, their performance was not correlated with each other. These results confirmed that children may employ different types of representation in remembering sets of dots in the WM Number Dot task and the WM Visual Dot task.

In Experiments 1 and 2, since we only tested 5‐year‐old children, this may not be sufficient to reveal any age‐related changes. To further examine the early developmental trajectories of children representing different types of stimuli in WM and the emergence of the role of knowledge contributing to WM performance when representing the conceptual information, in Experiment 3, we extended the age range to younger children, with a goal to examine the early developmental trends in remembering different types of information using the four WM tasks.

Furthermore, in our study, since we probed children's representation of conceptual information through examining children's extraction of numerical information from sets of dots, we targeted children between the ages of 3 and 5 years, as previous work showed that children's knowledge of numbers starts to grow and develop rapidly surrounding this age range (Wynn 1990; Schneider et al. 2021). For example, children typically become full counters or cardinal principal knowers between the ages of 4 and 5 (Sarnecka and Carey 2008; Wynn 1990), for whom the maturation of number knowledge may facilitate children to extract the numerical information of the dots more efficiently, therefore helping children to perform better in the WM Number Dot task compared to the WM Visual Dot task.

## 5 Experiment 3

### 5.1 Participants

Seventy‐seven 3‐year‐old children (mean age = 3.64 years, range: 3.01–3.98 years, 39 girls) and 88 four‐year‐old children (mean age = 4.44 years, range: 4.00–4.99 years, 45 girls) participated in this study. Children were assigned to one of the two groups, each completing two WM tasks. One group completed the WM Animal task and the WM Number Dot task (37 three‐year‐olds, mean age = 3.64 years, range: 3.17–3.98 years, 19 girls; 47 four‐year‐old children, mean age = 4.43 years, range: 4.00–4.99 years, 24 girls). The other group completed the WM Dot task and the WM Visual Dot task (40 three‐year‐olds, mean age = 3.65, range: 3.97–3.01 years, 20 girls; 41 four‐year‐olds, mean age = 4.45 years, range: 4.00–4.98 years, 21 girls). Additional nine children (6 three‐year‐olds and 3 four‐year‐olds) were recruited but excluded due to incomplete participation. The sample size was determined based on the G*Power results to reach 80% power with a medium effect size (*f^2^* = 0.15) in a linear multiple regression with 3 predictors (age, task type, and set size) and two random intercepts (Participant and Trial number), which suggests *n* = 77 for each group.

Data collection was conducted in the same kindergarten as in Experiments 1 and 2 in Shanghai, China. The demographic information on their parents was reported as follows: 87.9% of fathers have a bachelor's degree or higher, and 91.9% of mothers have a bachelor's degree or higher. Consent forms were obtained from participating children's parents or legal guardians prior to the data collection. The consent form has been approved by the ethical committee of the Shanghai Normal University.

### 5.2 Design

Considering younger children's limited attention span and constrictions in guaranteeing two consecutive visits within one month, we decided to adopt a between‐subject design, and each child participant completed two WM tasks. One group of children performed the WM Animal task and the WM Number Dot task, and the other group of children performed the WM Dot task and the WM Visual Dot task; the order of which task came first was counterbalanced across participants.

### 5.3 Apparatus, Stimuli, and Procedures

The apparatus, stimuli, and procedures of the four WM tasks were identical as in Experiments 1 and 2. All children completed the two tasks within one testing session. The total testing duration was approximately 20–30 min.

### 5.4 Results

To determine whether young children can perform the WM tasks, we first conducted one‐sample tests on 3‐ and 4‐year‐olds’ WM performance separately. To correct for multiple comparisons across set sizes, the alpha value was set to be 0.01 in the WM Animal task and 0.0167 in the other three WM tasks. A detailed summary can be found in the Supporting Information: Tables S4 and S5. Overall, we found that 3‐year‐old children only performed significantly above chance when there were two animal characters to be remembered, *t*(36) = 5.99, *p* < 0.001, Cohen's *d* = 2.00. All other types of trials in the four WM tasks were at chance level, all *t* < 2.60, *p* > 0.13, Cohen's *d* < 0.83. For 4‐year‐olds, children's memory performance was significantly above chance when remembering up to four animal items, all *t*(46) > 6.03, *p* < 0.001, Cohen's *d* > 1.78. Furthermore, we observed that 4‐year‐old children started to show above‐chance performance when there were two items to be remembered in the WM Dot task (*t*(40) = 4.48, *p* < 0.001, Cohen's *d* = 1.42) and WM Number Dot task (*t*(46) = 3.73, *p* < 0.001, Cohen's *d* = 1.10), but not in the WM Visual Dot task (*t*(40) = 2.37, *p* = 0.036, Cohen's *d* = 0.69). Performance in all other types of trials remained at chance.

**FIGURE 4:**
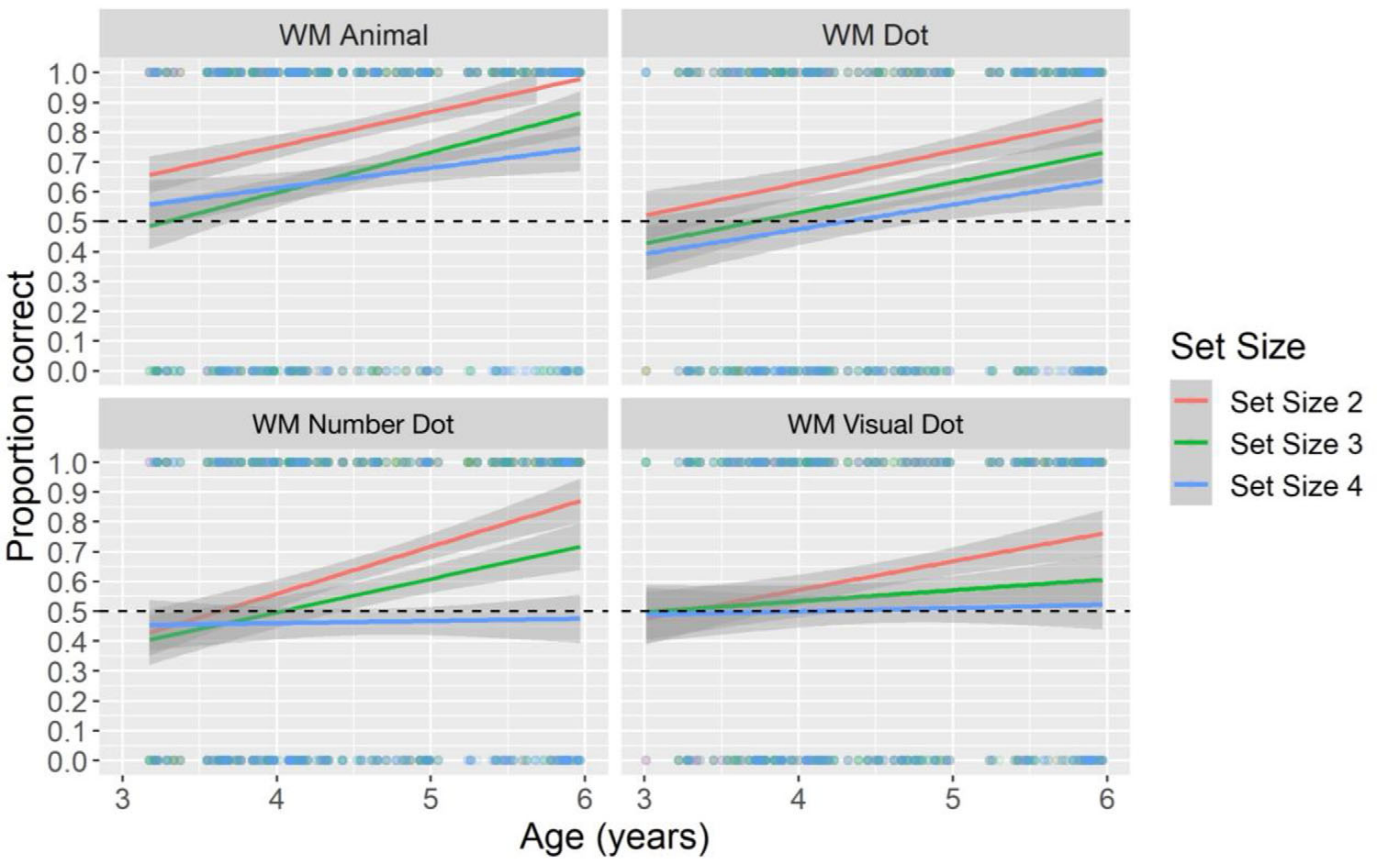
Comparison of children's memory performance over different set sizes in WM Animal task and WM Dot task (upper panel), and WM Number Dot task and WM Visual Dot task (lower panel) between the age of 3 and 6 years. The grey areas show the standard error.

To capture developmental changes of representing different types of content in WM from early to middle childhood, we combined 5‐year‐olds’ data from Experiment 1 and looked at how age, task type, and set size affect children's WM performance in representing images with conceptual and perceptual features in a generalized logistic regression using lme R package. We set Participant and Trial number as random intercepts. The best fitting model did not include any interactions between age, task, and set size. The model revealed a main effect of age (*F*(1, 182) = 77.40, *p* < 0.001, *η_p_* ^2^ = 0.30), task type (*F*(1, 2886) = 48.76, *p* < 0.001, *η_p_* ^2^ = 0.02), and set size (*F*(2, 2837) = 37.69, *p* < 0.001, *η_p_* ^2^ = 0.03). Children's memory performance in both WM Animal and WM Dot tasks increased with age. The performance in the WM Animal task was consistently higher than in the WM Dot task throughout the ages between 3 and 6. As the set size increased, children's performance decreased (see Figure 4, upper panel).

Next, we combined 5‐year‐old children's performance from Experiment 2 to examine the developmental factors, including age, task type, and set size, that may influence the memory performance in the WM Number Dot task and WM Visual Dot task from 3 to 6 years of age. We set Participant and Trial number as random intercepts. The best‐fitting model includes interactions between age, set size, and task type. The model showed a main effect of age (*F*(1, 173) = 36.59, *p* < 0.001, *η_p_* ^2^ = 0.17), task type (*F*(1, 2808) = 4.41, *p* = 0.036, *η_p_* ^2^ = 0.002), and set size (*F*(2, 2836) = 5.59, *p* = 0.004, *η_p_* ^2^ = 0.004). Children showed an age‐related increase in overall performance. We further observed an interaction effect between task type and age (*F*(2, 2792) = 4.46, *p* = 0.035, *η_p_* ^2^ = 0.002) and an interaction between set size and age (*F*(2, 2836) = 11.44, *p* < 0.001, *η_p_* ^2^ = 0.008). An inspection of Figure 4 showed that the performance for both tasks was similarly at chance when children were younger, at around age four, whereas the performance in the WM Number Dot task improved faster after 4.5 years old compared to the WM Visual Dot task, suggesting different developmental pace in remembering objects with different featural dimensions. Furthermore, with the increase in age, children's memory performance in remembering conceptual‐dominated features in Set Size 2 trials improved faster, whereas the memory performance for larger set sizes underwent a protracted development.

### 5.5 Discussion

The results of Experiment 3 provided insights into the developmental trajectory of WM in early childhood. The study examined how 3‐ to 5‐year‐old children processed different types of information—animal images and sets of dots in either or both conceptual and perceptual dimensions. Results revealed clear age‐related improvements in WM performance for all four tasks, particularly when remembering familiar animal images.

Comparing children's performance in the WM Animal task and the WM Dot task, children between the ages of 3 and 6 showed overall better performance in remembering animal pictures that are rich in both conceptual and perceptual dimensions, compared to pictures with dots. As expected, children's WM performance increased with age, and this developmental improvement was dependent on the set size. In the WM Animal task, statistical analyses (see details in the Supporting Information: Tables S4 and S5) showed that children as young as 3.5‐year‐olds can remember two cards with animal pictures. When children grow older, 4‐year‐olds start to be able to remember three to four cards. An inspection of Figure 4 suggested the age‐related improvement was faster in Set Size 3 than Set Size 4. Whereas children's WM performance in the WM Dot task developed later and slower compared to the WM Animal task. These results suggest that representing numerical dots posed additional cognitive resources in WM. One interpretation is that remembering animal images taps into semantic memory, enabling children to draw on long‐term representations of familiar objects. This may explain the relatively lower performance and slower developmental improvement in the WM Dot task compared to the WM Animal task.

Regarding children's performance in the WM Number Dot task and the WM Visual Dot task, we observed an interaction effect between task type and age, suggesting that the performance in remembering conceptual‐only information and perceptual‐only information of dotted images was developing at different paces over early childhood. When children were younger, their performance in the WM Number Dot task and WM Visual Dot task was not different from each other. However, with increases in age, the performance in representing the image's conceptual domain was developing faster and better compared to representing the image's perceptual domain, showing an age‐related interaction between the representational domains. In addition, the interaction effect between set size and age showed that while children's memory performance in remembering fewer items (e.g., two and three) in both tasks grows rapidly between the ages of 4 and 6 years, the expansion of memory loads significantly constrains children's memory performance. These findings further suggest that the WM capacity for representing either conceptual or perceptual information of dots is going through protracted development beyond the tested age range.

## 6 General Discussion

The present research provides a comprehensive examination of how different types of representational contents, namely, objects with conceptual and perceptual features, affect WM performance in early childhood. Across three experiments, we observed age‐related improvements in WM performance, with distinct differences in how children process and retain a variety of contents: animal images and sets of dots. These findings provide important insights into the nature of WM development, shedding light on how children represent different types of information in WM in early childhood.

Throughout the experiments, we consistently observed differences in WM performance when children were tasked to remember objects that can be encoded in conceptual aspect only (WM Number Dot task), perceptual aspect only (WM Visual Dot task), and objects that can be encoded in both conceptual and perceptual dimensions (WM Animal task and WM Dot task) in WM. These findings suggest that different types of WM stimuli could result in very different levels of performance (e.g., Brady et al. 2016). Children performed better on tasks involving meaningful, semantically rich stimuli, such as animal pictures used in the WM Animal task, compared to the dotted pictures in the WM Dot task. Results of Experiment 3 further provided a complementary developmental picture on the age‐related improvements in WM performance, consistent with previous work (Cowan 2016; Cheng and Kibbe 2022; Riggs et al. 2006; Simmering 2012). Younger children demonstrated a more limited capacity in WM, particularly in tasks that involve remembering sets of dots, in which the stimuli were not rich in either conceptual or perceptual dimension. As children grew older, their WM capacity expanded; this expansion was observed faster in tasks where children could integrate both conceptual and perceptual information to enhance their memory performance across all tested set sizes. By age 4, children could remember more items in the WM Animal, and they could also remember up to two sets of dots, though their performance remained higher with animal stimuli. This observation is consistent with Brady et al.’s (2019) findings, which indicate that children's ability to employ rich semantic information significantly enhances their memory performance. The age‐related improvements observed in the WM Animal task suggest that children's ability to use semantic information—drawing on long‐term memory and prior knowledge—plays a critical role in how they manage WM demands.

While it is known that different types of stimuli may result in different levels of memory performance, the current study was the first to directly compare the role of representational content, while controlling for the perceptual similarity, in the development of WM capacity in early childhood. This was particularly important for young learners with rapid enrichment and expansion in their knowledge domains, in which conceptual information becomes more prioritized in information processing. In our study, by comparing young children's WM performance in representing either the perceptual or the conceptual aspects of the stimuli, our results showed that conceptual representation may be more beneficial in remembering stimuli in WM compared to representing the perceptual dimension, as it allows children to leverage existing cognitive structures, such as semantic knowledge and categorization, to support their representational precision in WM. In contrast, when focusing only on the perceptual dimension, like in the WM Visual Dot task, the layout of the dots brought little conceptual meaning. Children can only focus on the visual‐spatial dimension of the stimuli, which prevents them from integrating conceptual meaningful numerical information into WM. This finding aligns with previous work showing that conceptual categorization enhances WM performance by integrating conceptual information into the representation, which creates a more robust representation in WM (e.g., Brady et al. 2013; Feigenson and Halberda 2008; Kibbe and Leslie 2019). Focusing on a single dimension, particularly in the visual‐spatial dimension only, in contrast, poses a more limited capacity for retaining abstract visual information.

While children performed better in the WM Number Dot task compared to the WM Visual Dot task when remembering fewer than four items, we observed that their performance was susceptible to memory loads. With the increase in set size, children's memory performance declined significantly when there were four cards to remember, bringing the performance to chance level, similar to the performance in the WM Visual Dot task. Converging evidence was shown in Experiment 3 that while children's WM performance increased with age in Set Sizes 2 and 3, their performance in Set Size 4 was consistently at chance throughout the tested age range. This suggested that higher memory loads posed a significant barrier in both conceptualizing the numerical meanings as well as remembering the visual‐spatial orientation of the dots, and improvement in larger set sizes may undergo a protracted development beyond age six. One possibility is that children may not automatically subitize four dots as “four,” and this subitizing ability may not be directly linked with the age‐related increase in the acquisition of number knowledge. Instead, processing the number of dots may take up additional perceptual resources, highlighting the need for further investigation in future studies. It also provides empirical implications for developmental researchers to consider both the memory content and its capacity limitation when designing cognitive tasks that involve different types of stimuli.

The results of this study have practical implications, particularly in early childhood education. Educators can use these findings to tailor learning environments that align with children's cognitive abilities. For instance, materials that leverage children's ability to process and retain semantically meaningful information, such as images of familiar objects or categories, can enhance memory retention and reduce cognitive loads. Similarly, when educators use non‐symbolic formats to demonstrate mathematically related concepts, educators should be mindful of children's WM capacity when processing numerically relevant stimuli. By structuring learning around conceptually rich stimuli and other visual and spatial scaffolding, educators can help children develop better memory strategies and manage cognitive tasks more efficiently. Meanwhile, tasks that rely on abstract and perceptual information should be introduced more gradually, with careful scaffolding to ensure that children are not overwhelmed by cognitive loads. For example, visual‐spatial tasks that require children to discriminate between similar abstract shapes should be broken down into simpler steps to help children develop the necessary visual‐spatial processing skills without overtaxing their WM capacity.

Our study has certain limitations that pave the way for future research opportunities. First, in the present study, we used sets of dots as a case study to examine how children's WM performance varies when representing conceptual, perceptual, or both dimensions of stimuli. This approach allowed us to investigate memory representations across different processing dimensions. However, the differences in performance between the WM Animal task and the WM Dot task may also be influenced by children's varying levels of numerical skills, rather than solely by the reduced richness of the conceptual and perceptual dimensions. While exploring the factors that may affect children's processing of numerical information in sets of dots was beyond the scope of the present study, future research is needed to address this question. One potential future direction is to explicitly examine the role of numerical knowledge in the development of the ability to remember sets of dots. This could involve measuring individual participants’ numerical skills and comparing children's performance across different WM tasks. Another avenue for future research is to use alternative types of stimuli to examine WM capacity for representing conceptual versus perceptual information beyond dots. Such research could provide additional evidence across other knowledge domains and help evaluate the generalizability of the present findings.

## Author Contributions

**Tongyan Ren**: investigation, writing – original draft, writing – review and editing, project administration. **Xuechen Ding**: supervision, funding acquisition, investigation, resources, writing – review and editing. **Chen Cheng**: conceptualization, methodology, supervision, formal analysis, visualization; writing – original draft, writing – review and editing.

## Ethics Statement

The study protocol was approved by the ethical committee board of the Shanghai Normal University.

## Conflicts of Interest

The authors declare no conflicts of interest.

## Supporting information




**Supporting File 1**: desc70066‐sup‐0001‐SuppMat.docx

## Data Availability

The stimuli used in the study was available on OSF at https://osf.io/m4fbq. The data is available upon request from the authors.
