# Supplementary material for: A Dotted Triangle or Dots of Three: The Role of Representational Content on Working Memory Capacity in Early Childhood
Source: Dev Sci. 2025 Aug 18;28(5):e70066. doi: 10.1111/desc.70066 (PMC12361590; doi:10.1111/desc.70066)
Supplement: Supplementary file 1 — Supporting File 1: desc70066‐sup‐0001‐SuppMat.docx [file DESC-28-e70066-s001.docx]

|  |  | Mean (SD) | *t*(*39*) | *p* | *Cohen's d* |
| --- | --- | --- | --- | --- | --- |
| WM Animal task | Set size 2 | 0.97 (0.84) | 35.40 | <0.001 | 11.34 |
|  | Set size 3 | 0.80 (0.21) | 8.90 | <0.001 | 2.85 |
|  | Set size 4 | 0.71 (0.23) | 5.84 | <0.001 | 1.87 |
|  | Set size 5 | 0.68 (0.24) | 4.58 | <0.001 | 1.47 |
|  | Set size 6 | 0.56 (0.29) | 1.35 | 0.19 | 0.43 |
| WM Dot task | Set size 2 | 0.83 (0.22) | 9.43 | <0.001 | 3.02 |
|  | Set size 3 | 0.74 (0.28) | 5.42 | <0.001 | 1.74 |
|  | Set size 4 | 0.66 (0.26) | 3.75 | <0.001 | 1.20 |

**Supplemental Material**

Table S1. Details of one-sample t tests results in Experiment 1.

|  | |  | Mean (SD) | *t*(*39*) | *p* | *Cohen's d* |
| --- | --- | --- | --- | --- | --- | --- |
| WM Number Dot task | Set size 2 | | 0.84 (0.20) | 10.64 | <0.001 | 3.41 |
|  | Set size 3 | | 0.71 (0.25) | 5.30 | <0.001 | 1.70 |
|  | Set size 4 | | 0.47 (0.27) | 0.73 | 0.47 | 0.23 |
| WM Visual Dot task | Set size 2 | | 0.74 (0.22) | 6.92 | <0.001 | 2.22 |
|  | Set size 3 | | 0.64 (0.18) | 4.87 | <0.001 | 1.56 |
|  | Set size 4 | | 0.54 (0.25) | 1.13 | 0.27 | 0.36 |

Table S2. Details of one-sample t tests results in Experiment 2.

|  | WM Animal | WM Dot task | WM Number Dot task |
| --- | --- | --- | --- |
| WM Animal task | - | - | - |
| WM Dot task | .498^**^  (.001) | - | - |
| WM Number Dot task | .360^*^  (.024) | .568^**^  (<.001) | - |
| WM Visual Dot task | .476^**^  (.002) | .378^*^  (.018) | .263  (.105) |

Table S3. Partial correlational results, controlling for age.

| Task | Set Size | Mean (SD) | *t* | *p* | *Cohen's d* |
| --- | --- | --- | --- | --- | --- |
| WM Animal task | Set size 2 | 0.72 (0.23) | 5.99 | <0.001 | 2.00 |
|  | Set size 3 | 0.49 (0.22) | 0.18 | 0.86 | 0.06 |
|  | Set size 4 | 0.55 (0.27) | 1.21 | 0.23 | 0.40 |
|  | Set size 5 | 0.47 (0.23) | 0.70 | 0.49 | 0.23 |
|  | Set size 6 | 0.47 (0.23) | 0.70 | 0.49 | 0.23 |
| WM Dot task | Set size 2 | 0.61(0.26) | 2.60 | 0.13 | 0.83 |
|  | Set size 3 | 0.54 (0.18) | 1.29 | 0.21 | 0.41 |
|  | Set size 4 | 0.47 (0.21) | 0.93 | 0.36 | 0.30 |
| WM Number Dot task | Set size 2 | 0.53 (0.46) | 0.45 | 0.66 | 0.15 |
|  | Set size 3 | 0.45 (0.28) | 1.19 | 0.24 | 0.40 |
|  | Set size 4 | 0.47 (0.22) | 0.92 | 0.36 | 0.31 |
| WM Visual Dot task | Set size 2 | 0.57 (0.29) | 1.51 | 0.14 | 0.48 |
|  | Set size 3 | 0.53 (0.28) | 0.56 | 0.58 | 0.18 |
|  | Set size 4 | 0.53 (0.21) | 0.93 | 0.36 | 0.30 |

Table S4. Details of one-sample t tests results in 3-year-olds of Experiment 3.

| Task | Set Size | Mean (SD) | *t* | *p* | *Cohen's d* |
| --- | --- | --- | --- | --- | --- |
| WM Animal task | Set size 2 | 0.79 (0.20) | 9.83 | <0.001 | 3.28 |
|  | Set size 3 | 0.73 (0.20) | 8.11 | <0.001 | 2.70 |
|  | Set size 4 | 0.69 (0.21) | 6.03 | <0.001 | 2.01 |
|  | Set size 5 | 0.53 (0.19) | 1.14 | 0.26 | 0.38 |
|  | Set size 6 | 0.54 (0.22) | 1.31 | 0.2 | 0.44 |
| WM Dot task | Set size 2 | 0.65 (0.21) | 4.48 | <0.001 | 1.42 |
|  | Set size 3 | 0.50 (0.26) | 0.00 | 1.00 | <.001 |
|  | Set size 4 | 0.45 (0.21) | 1.48 | 0.15 | 0.47 |
| WM Number Dot task | Set size 2 | 0.65 (0.28) | 3.73 | <0.001 | 1.24 |
|  | Set size 3 | 0.55 (0.22) | 1.65 | 0.11 | 0.55 |
|  | Set size 4 | 0.45 (0.23) | 1.42 | 0.16 | 0.47 |
| WM Visual Dot task | Set size 2 | 0.58 (0.23) | 2.17 | 0.036 | 0.69 |
|  | Set size 3 | 0.51 (0.22) | 0.18 | 0.86 | 0.06 |
|  | Set size 4 | 0.45 (0.22) | 1.65 | 0.11 | 0.52 |

Table S5. Details of one-sample t tests results in 4-year-olds of Experiment 3.
